# A highly powerful nonspecific strategy to reduce COVID‐19 deaths

**DOI:** 10.1002/jmv.27949

**Published:** 2022-07-01

**Authors:** Ji‐Ming Chen, Guo‐Hui Li, Yu‐Fei Ji, Ming‐Hui Sun, Huan‐Yu Gong, Rui‐Xu Chen, Ji‐Wang Chen

**Affiliations:** ^1^ School of Life Science and Engineering Foshan University Foshan China; ^2^ Division of Pulmonary, Critical Care, Sleep, and Allergy, Department of Medicine University of Illinois at Chicago Chicago Illinois USA

**Keywords:** case fatality rate, co‐infection, COVID‐19, pandemic, SARS‐CoV‐2, strategy

## Abstract

The coronavirus disease 2019 (COVID‐19) pandemic caused by the coronavirus severe acute respiratory syndrome coronavirus 2 remains risky worldwide. We elucidate here that good IDM (isolation, disinfection, and maintenance of health) is powerful to reduce COVID‐19 deaths based on the striking differences in COVID‐19 case fatality rates among various scenarios. IDM means keeping COVID‐19 cases away from each other and from other people, disinfecting their living environments, and maintaining their health through good nutrition, rest, and treatment of symptoms and pre‐existing diseases (not through specific antiviral therapy). Good IDM could reduce COVID‐19 deaths by more than 85% in 2020 and more than 99% in 2022. This is consistent with the fact that good IDM can minimize co‐infections and maintain body functions and the fact that COVID‐19 has become less pathogenic (this fact was supported with three novel data in this report). Although IDM has been frequently implemented worldwide to some degree, IDM has not been highlighted sufficiently. Good IDM is relative, nonspecific, flexible, and feasible in many countries, and can reduce deaths of some other relatively mild infectious diseases. IDM, vaccines, and antivirals aid each other to reduce COVID‐19 deaths. The IDM concept and strategy can aid people to improve their health behavior and fight against COVID‐19 and future pandemics worldwide.

## Highlights

- Multiple striking differences in COVID‐19 case fatality rate were calculated.
- These differences support that a strategy could reduce COVID‐19 deaths by more than 85% in 2020 and more than 99% in 2022.
- The strategy is based on isolation, disinfection, and health maintenance.
- The strategy can reduce co‐infections and maintain body functions.
- The strategy has been implemented frequently but insufficiently worldwide.
- The strategy is non‐specific and can reduce deaths of other mild infectious diseases.

## 1 INTRODUCTION

The coronavirus disease 2019 (COVID‐19) pandemic caused by severe acute respiratory syndrome coronavirus 2 (SARS‐CoV‐2) has led to 6.32 million deaths worldwide and remains risky to many people worldwide.^1^, ^2^ Unlike in 2020, now COVID‐19 vaccines and specific antivirals, such as Molnupiravir and Paxlovid, can all reduce dramatically COVID‐19 severe cases and deaths.^1^, ^2^ We elucidate here another strategy that is highly effective to reduce COVID‐19 deaths. This strategy is termed IDM (isolation, disinfection, and maintenance of health), which means keeping COVID‐19 cases away from each other and from other people, disinfecting their living environments, and maintaining their health through good nutrition, good rest, and treatment of symptoms and pre‐existing diseases without specific effective antivirals. IDM covers various non‐pharmaceutical interventions and pharmaceutical interventions.

In principle, good IDM can reduce COVID‐19 deaths because it can minimize co‐infections and maintain body functions to fight against various diseases. It has been found that co‐infection with influenza and other pathogens increased substantially COVID‐19 case fatality rates (CFRs),^3^ and shortage of medical services (e.g., providing with ventilators) to maintain the health of COVID‐19 cases accounted for vast COVID‐19 deaths.^4^

IDM has been implemented worldwide to some degree and its principle is comprehensible, but the integration of isolation of COVID‐19 cases, disinfection of their living environments, and maintenance of their health into the IDM concept is new. Moreover, it remains unknown about the effectiveness of IDM to reduce COVID‐19 deaths and IDM has not been highlighted sufficiently.

**Figure 1:**
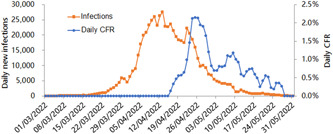
Daily new coronavirus disease 2019 (COVID‐19) infections and case fatality rate (CFR) in Shanghai in 3‐day average from March to May of 2022. Rapid increase of new COVID‐19 infections in April 2022 worsened the isolation, disinfection, and maintenance of health (IDM) of COVID‐19 cases because many COVID‐19 cases had to live together in temporary hospitals which were less comfortable than their homes and created co‐infection opportunities.

**Figure 2:**
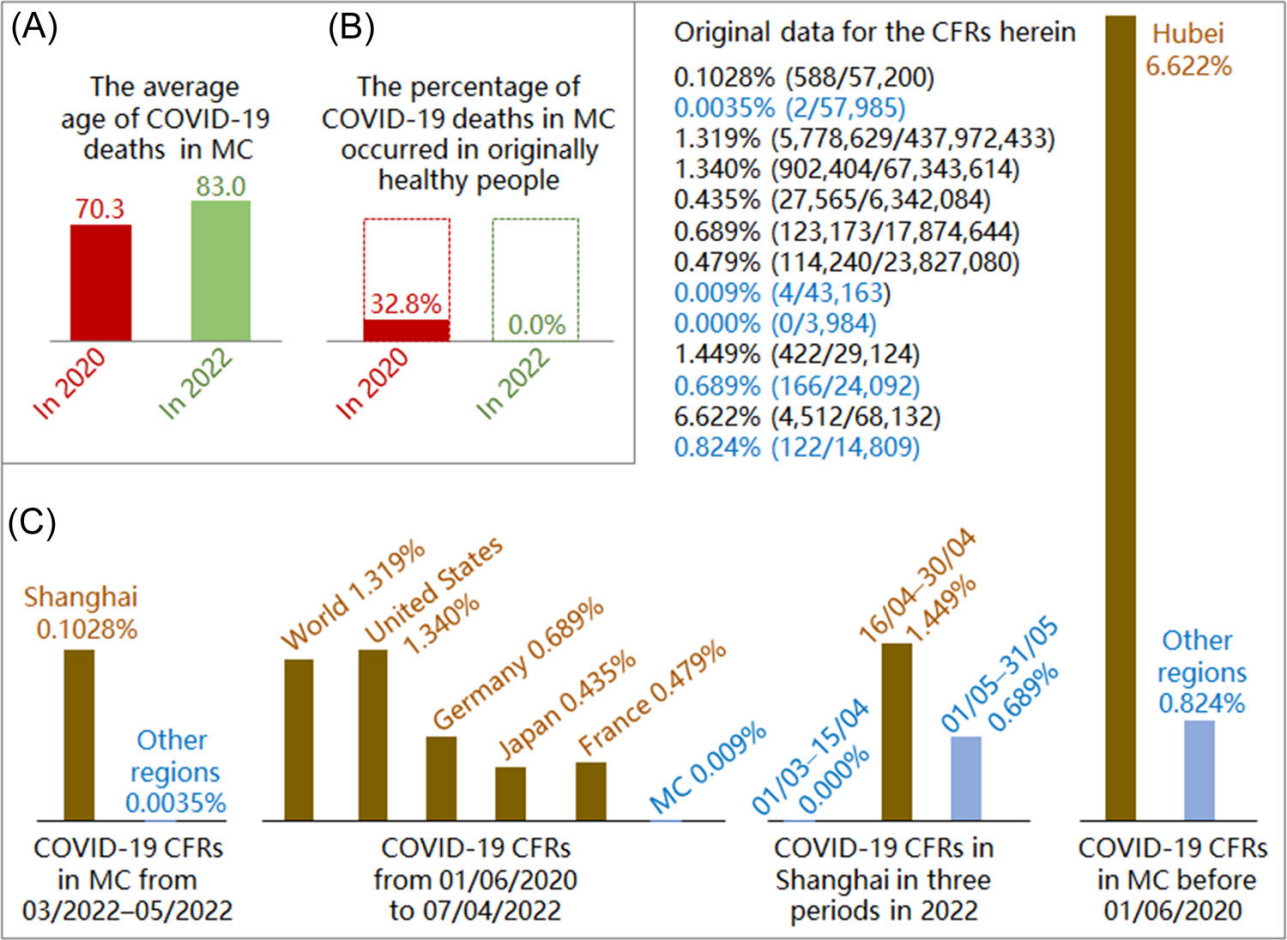
Differences in the average ages and original health of the first hundreds of coronavirus disease 2019 (COVID‐19) deaths in mainland China (MC) in 2020 and 2022 (A and B) and differences in COVID‐19 case fatality rates (CFRs) in various scenarios (C). The blue data and pillars in panel C represented the scenarios with better isolation, disinfection, and maintenance of health (IDM) in the relevant groups.

Numerous COVID‐19 cases in many countries or regions outside mainland China (MC) could go out to meet other people, live in rooms without disinfection, or be too poor to maintain their health. By contrast, to block completely COVID‐19 transmission in MC which has stuck to the strict zero‐COVID policy since January 2020, each COVID‐19 case in MC was usually separated in a single room with good disinfection and good health maintenance, except in the following two scenarios. One was in Hubei province before June 2020 because too many severe COVID‐19 cases in Hubei in that period led to shortage of medical services. The other was in Shanghai in April 2022 because too many asymptomatic or mild COVID‐19 cases in the city in that period had to live together in temporary hospitals to separate them from uninfected people, which created co‐infection opportunities and led to poor maintenance of health for elders with comorbidities (Figure 1).^5^, ^6^, ^7^, ^8^ For example, on April 26, 2022, around 45 000 asymptomatic or mild COVID‐19 cases including approximately 6500 elders aged ≥70 lived in the same temporary hospital established from National Convention and Exhibition Center (Shanghai). Some elders with comorbidities encountered difficulties in this temporary hospital.^9^ Of the first 65 000 people infected with COVID‐19 discharged from this temporary hospital, 20 became severe cases due to aggravation of their comorbidities, and none became severe cases due to viral pneumonia caused by SARS‐CoV‐2.^9^ Together, the world created 13 scenarios in four groups with different IDM to fight COVID‐19 (Figure 2), which could be employed to investigate the effectiveness of IDM to reduce COVID‐19 deaths.

## 2 METHODS AND MATERIALS

Information of COVID‐19 deaths in MC and counts of COVID‐19 infections, cases, deaths, and recoveries of various countries or regions were collected from the relevant official websites or reports.^1^, ^5^, ^6^, ^7^, ^10^ They were partially shown in Figure 2. COVID‐19 CFRs were calculated as per the standard method by dividing the relevant death counts by the counts of cases with known consequences (death or recovery). Daily COVID‐19 CFRs in a 3‐day average were calculated by dividing the death counts of the relevant 3 days (Days −1, 0, and 1) by the counts of cases with known consequences of the same 3 days. The average death age was calculated with daily deaths counts and daily average death ages for 2022, and estimated with the products of the deaths counts of each age group and the median of the age group for 2020 (the median of the age group of 80 plus was estimated as 90). Dates were expressed in dd/mm/yy.

## 3 RESULTS

Figure 2A showed that in 2022 the average age of the first 590 COVID‐19 deaths in MC (588 in Shanghai and 2 in Jilin) was 83.0, 5.5 years higher than the life expectancy 77.5 in MC in 2020.^11^ All these 590 people died directly of their severe comorbidities rather than COVID‐19.^5^, ^6^, ^7^ By contrast, in 2020, the average age of the first 1023 COVID‐19 deaths in MC was estimated to be 70.3, and 32.8% of them were originally healthy without comorbidities (Figure 2B).^10^ These data suggest SARS‐CoV‐2 has been much less pathogenic in 2022, which is consistent with previous reports.^2^, ^12^

Figure 2C showed that COVID‐19 CFR was strikingly higher in all the scenarios with worse IDM (*p* < 0.01, by Chi‐square test). COVID‐19 CFR was higher by 297 times in Shanghai than in other regions of MC from March 1 to May 31, 2022, by 46−145 times higher in various countries or regions than in MC from June 1, 2020 to April 7, 2022, and by 7 times in Hubei province than in other provinces of MC before June, 2020. Consistent with Figure 1, COVID‐19 CFR in Shanghai was 0.000% from March 1, 2022 to April 15, 2022, 1.449% from April 15, 2022 to April 30, 2022, and then 0.689% in 05/2022. These striking differences in COVID‐19 CFRS all support that good IDM is highly effective to reduce COVID‐19 deaths. Meanwhile, the CFR in MC with good IDM was higher by 238 times in 2020 (0.824%) than in 2022 (0.0035%) (Figure 2C), which further supported that COVID‐19 has become less pathogenic in 2022.

Good IDM could reduce COVID‐19 deaths by more than 99% in 2022 from the COVID‐19 CFRs of Shanghai in March and April of 2022 (0.000% versus 1.449%) and by more than 85% in 2020 from the COVID‐19 CFRs in MC before June 2020 (6.622% versus 0.824%) (Figure 2C).

## 4 DISCUSSION

MC has successfully maintained its strict zero‐COVID policy from January 2020. To block completely COVID‐19 transmission, all the people infected with COVID‐19 in MC were identified and their recent traveling histories were published as soon as possible, and their outcomes of recovery or death were known to many people.^5^, ^6^, ^7^ Therefore, the reported data of COVID‐19 infections, cases, recoveries, and deaths in MC were all reliable and nearly equal to their truth values. An analysis suggested that, besides MC, COVID‐19 data of Japan, France, Germany, and the United States were also relatively reliable, so their CFRs were employed in this study (Figure 2).^13^

Various factors, such as data quality, case definition, country, region, city, climate, pollution, virus evolution, human race, smoking, drinking, vaccination coverage, population youngness, or medications, could account partially for some striking differences in COVID‐19 CFRS listed in Figure 2.^14^, ^15^ None of them could account for all the above striking differences, and none of them could account for the COVID‐19 CFR differences in the same city of Shanghai between the three adjacent periods (Figures 1 and 2), except IDM. Meanwhile, we created the IDM concept because we cannot differentiate the effectiveness of the three parts of IDM to reduce COVID‐19 deaths through these striking differences in COVID‐19 CFRs.

In principle, good IDM is more effective to reduce deaths of mild infectious diseases (e.g., influenza) than to reduce deaths of highly pathogenic infectious diseases (e.g., rabies). This is consistent with the above results that good IDM was more effective to reduce COVID‐19 deaths in 2022 than in 2020 and that COVID‐19 has become less pathogenic in 2022 than in 2020.

Good IDM is more crucial to those unvaccinated elders with comorbidities than to others because these elders are the most vulnerable to COVID‐19 infection. Good IDM is more crucial in the regions or periods with co‐circulation of influenza, respiratory syncytial virus, or another respiratory pathogen than in other regions or periods.

Good IDM is usually relatively good. For example, in isolation, staying at home is better than shopping which is better than dancing in a crowded room for hours. In disinfection, well‐disinfected rooms are better than clean rooms without disinfection which is better than dirty rooms.

Good IDM is flexible, and various behavior or measures belong to good IDM if they have the same functions of IDM in minimizing co‐infections and maintaining body functions. For example, walking out for a while with social distancing belongs to good IDM for mild cases. Vaccination for influenza can be an important IDM measure for COVID‐19 and has been shown highly effective to reduce COVID‐19 deaths in young people in an influenza season in Qatar,^16^ likely because influenza vaccination can minimize co‐infection of COVID‐19 cases with influenza.

Good IDM is nonspecific and can reduce deaths of other mild infectious diseases. Therefore, one can manage good IDM in his home after he has shown influenza‐like symptoms without clear diagnosis. Good IDM is hence feasible in many countries with or without mass COVID‐19 detection capacity. Meanwhile, good IDM is usually less costly for acute self‐limited infectious diseases (e.g., influenza and COVID‐19) than for chronic infectious diseases (e.g., hepatitis B and AIDs).

Usually, mild cases can manage good IDM in their homes because their health is relatively easy to be well maintained.^17^ Severe cases can usually obtain good IDM in hospitals, although patients in some hospitals may also encounter poor IDM if the hospitals are full of patients and nosocomial infections are not well controlled.

Good IDM is significantly affected by many factors such as healthy policies, regulations, education, culture, economics, and individual behavior (e.g., smoking and exercising). Therefore, the guidance of good IDM can be different for different countries, regions, and individuals. Usually, people have to balance good IDM versus freedom, legal rights, and economy.

IDM can explain some elusive phenomena, such as the above striking and elusive differences in COVID‐19 CFRs and the high effectiveness of influenza vaccination to reduce COVID‐19 deaths.^16^, ^17^, ^18^ Moreover, good IDM is feasible and flexible in many countries to reduce deaths of relatively mild infectious diseases. The IDM concept and strategy can aid people to improve their health behavior and defeat COVID‐19 and future pandemics.

**Figure 3:**
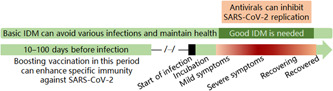
The targets and favorable usage time of vaccines, antivirals, and IDM. COVID‐19, coronavirus disease 2019; IDM, isolation, disinfection, and maintenance of health; SARS‐CoV‐2, severe acute respiratory syndrome coronavirus 2

Nonspecific good IDM, specific effective vaccines, and specific effective antivirals aid each other to reduce COVID‐19 deaths. They work on different targets (Figure 3). Those who are qualified in health for vaccination should be vaccinated with COVID‐19 vaccines in time, and all vaccinated or unvaccinated people should manage good IDM for themselves if they have influenza‐like symptoms, and severe COVID‐19 cases, no matter whether they have been vaccinated or obtained good IDM, should be treated with effective antivirals, if possible.^18^ Importantly, we should not just rely on good IDM to reduce the severity of rabies and other highly pathogenic infectious diseases. Meanwhile, some basic IDM measures (e.g., timely hand washing, avoiding being too tired or cold, and treatment of diabetes and other chronic diseases) should be considered to avoid various infections and maintain health (Figure 3).

## 5 CONCLUSION

We calculated multiple striking differences in COVID‐19 CFRS in various scenarios. These differences support that good IDM is highly powerful to reduce COVID‐19 deaths. Good IDM could reduce COVID‐19 deaths by more than 85% in 2020 and more than 99% in 2022. This is consistent with the theoretic functions of good IDM and the fact that COVID‐19 has been less pathogenic. Good IDM is relative, nonspecific, flexible, and feasible in many countries, and can reduce deaths of some other relatively mild infectious diseases. IDM, vaccines, and antivirals aid each other to reduce COVID‐19 deaths, and they cannot replace each other. The IDM concept and strategy can aid people to improve their health behavior and fight against COVID‐19 and future pandemics worldwide.

## AUTHOR CONTRIBUTIONS

Ji‐Ming Chen conceived, designed, and supported this study, analyzed the data, and drafted the manuscript. Ji‐Wang Chen made the core conclusion. All authors collected relevant data and revised the manuscript.

## CONFLICT OF INTEREST

The authors declare no conflict of interest.

## Data Availability

The data supporting the views of this analysis are available from the corresponding author on request.
